# Statistical modeling for sensitive detection of low-frequency single nucleotide variants

**DOI:** 10.1186/s12864-016-2905-x

**Published:** 2016-08-22

**Authors:** Yangyang Hao, Pengyue Zhang, Xiaoling Xuei, Harikrishna Nakshatri, Howard J. Edenberg, Lang Li, Yunlong Liu

**Affiliations:** 1 Department of Medical and Molecular Genetics, Indiana University School of Medicine, Indianapolis, IN 46202 USA; 2 Center for Computational Biology and Bioinformatics, Indiana University School of Medicine, Indianapolis, IN 46202 USA; 3 Department of Biostatistics, Indiana University School of Medicine, Indianapolis, IN 46202 USA; 4 Department of Biochemistry and Molecular Biology, Indiana University School of Medicine, Indianapolis, IN 46202 USA; 5 Center for Medical Genomics, Indiana University School of Medicine, Indianapolis, IN 46202 USA; 6 Department of Surgery, Indiana University School of Medicine, Indianapolis, IN 46202 USA; 7 IU Simon Cancer Center, Indiana University School of Medicine, Indianapolis, IN 46202 USA

## Abstract

**Background:**

Sensitive detection of low-frequency single nucleotide variants carries great significance in many applications. In cancer genetics research, tumor biopsies are a mixture of normal and tumor cells from various subpopulations due to tumor heterogeneity. Thus the frequencies of somatic variants from a subpopulation tend to be low. Liquid biopsies, which monitor circulating tumor DNA in blood to detect metastatic potential, also face the challenge of detecting low-frequency variants due to the small percentage of the circulating tumor DNA in blood. Moreover, in population genetics research, although pooled sequencing of a large number of individuals is cost-effective, pooling dilutes the signals of variants from any individual. Detection of low frequency variants is difficult and can be cofounded by sequencing artifacts. Existing methods are limited in sensitivity and mainly focus on frequencies around 2 % to 5 %; most fail to consider differential sequencing artifacts.

**Results:**

We aimed to push down the frequency detection limit close to the position specific sequencing error rates by modeling the observed erroneous read counts with respect to genomic sequence contexts. 4 distributions suitable for count data modeling (using generalized linear models) were extensively characterized in terms of their goodness-of-fit as well as the performances on real sequencing data benchmarks, which were specifically designed for testing detection of low-frequency variants; two sequencing technologies with significantly different chemistry mechanisms were used to explore systematic errors. We found the zero-inflated negative binomial distribution generalized linear mode is superior to the other models tested, and the advantage is most evident at 0.5 % to 1 % range. This method is also generalizable to different sequencing technologies. Under standard sequencing protocols and depth given in the testing benchmarks, 95.3 % recall and 79.9 % precision for Ion Proton data, 95.6 % recall and 97.0 % precision for Illumina MiSeq data were achieved for SNVs with frequency > = 1 %, while the detection limit is around 0.5 %.

**Conclusions:**

Our method enables sensitive detection of low-frequency single nucleotide variants across different sequencing platforms and will facilitate research and clinical applications such as pooled sequencing, cancer early detection, prognostic assessment, metastatic monitoring, and relapses or acquired resistance identification.

**Electronic supplementary material:**

The online version of this article (doi:10.1186/s12864-016-2905-x) contains supplementary material, which is available to authorized users.

## Background

In 2005, the first next-generation sequencing (NGS) technology was released by 454 Life Sciences (now Roche) [1]. Within the past ten years, different sequencing technologies and platforms, including Illumina, SOLiD, Ion Torrent, Complete Genomics, were released to the public. The much faster sequencing speed, high-throughput capacity and now up to several hundred bases read length, together with a greatly reduced cost, revolutionized the scope and efficiency of biomedical related field researches [2]. Paired with the increasingly diverse range of biological application of NGS technologies, numerous computational and informatics tools, frameworks and pipelines emerged to enable researchers to harness the power of NGS technologies. Statistical models suitable for count data modeling gained much attention in NGS data analysis due to the discrete count nature of the data generated by NGS sequencers. Such models were broadly applied in DNA sequencing (DNA-Seq) based variants identification such as samtools [3], VarScan2 [4], and SNVMix [5]. For DNA sequencing based single nucleotide variant (SNV) identification, emerging new applications bring challenges to refine the statistical modeling methods and pushing the limit of NGS technologies.

In cancer genetics research, low frequency tumor somatic SNV identification is crucial due to the inevitable normal tissue contamination [6, 7] and the highly heterogeneous, constantly evolving nature of tumors [8]. Accurate and sensitive identification of low frequency SNVs also carries clinical significance, since it enables the early diagnosis, cancer progression monitor and relapse identification. The recent discovery of circulating tumor DNA (ctDNA) also gained much attention. Contrast to traditional tumor biopsies, which is invasive and can only offer a snapshot of the tumor genetics landscape at certain checkpoints, ctDNA based ‘liquid biopsy’ [9] is non-invasive and can be done repeatedly for close monitoring of early sign of relapse or metastasis. However, ctDNA only takes a small percentage of all blood sample DNA, a previous research [10] reported for some advanced cancers, ctDNA is about 1 ~ 10 % of blood DNA.

The difficulty for low-frequency SNV identification using NGS technologies is due to the relatively high sequencing artifacts or error rates, which is around 0.1 ~ 1 % for most platforms. Further, such error rates differ significantly under various genome contexts. For example, Illumina sequencing data are prone to have mismatches while Ion Torren and Ion Proton data contain more homopolymer related indels and consequently, mismatches near homopolymer loci [11–13]. For somatic SNV identification paired tumor-normal design, some existing methods derive the sequencing error probability from base qualities followed by error likelihood ratio test of tumor and normal sample at the same location, for example in Mutect [7], Strelka [14]. While VarScan2 applies a Fisher’s exact test on the paired samples, treating non-reference read counts from the normal sample as background error rate. The former failed to consider differential error rates for substitution types while the latter only utilized information in one location thus the background error rate estimation is off. For one sample low-frequency SNV calling, UDT-Seq [15] tabulated the error rate based on substitution types, strand and location on the read to derive an empirical background error rate, then use binomial model to distinguish signal from error, and the candidate SNVs are further refined by 7 filters. This method is context-aware but also ad-hoc, thus the ability to adapt to different sequencing technologies is limited. A brief summary of the tools mentioned above is included in Additional file 1.

By analyzing previous efforts, our group proposed a framework (Yangyang Hao XX, Li L, Nakshatri H, Edenberg HJ, Liu Y. RareVar: A Framework for Detecting Low Frequency Single Nucleotide Variants, submitted) to first generated position specific error model (PSEM) using genome sequence contexts for candidate SNV identification and then apply a machine-learning model to refine the candidates. Testing on an Ion Proton benchmark dataset, our framework outperforms existing methods, especially at 0.5 % to 3 % frequencies. However, the potential to improve PSEM performances on SNVs with close to sequencing error rates by implementing more sophisticated statistical modeling and the generalizability and adaptiveness of PSEM remain untested. In this research, we explored what distributions fit the DNA-Seq data error rates modeling as well as the possibility of improved position specific error rate prediction for higher precision and recall on SNVs down to 0.5 % frequency. Further, we evaluated how different sequencing technologies affect the behavior of PSEM and the generalizability and adaptiveness of the PSEM framework.

## Results

In the Result section, we first briefly summarized the benchmark datasets used for PSEM. Then we compared the testing benchmark dataset from Ion Proton with Illumina MiSeq sequencing data in terms of allele frequency composition and depth distribution. Utilizing count data visualization plots and tabulation, we selected the candidate distributions that may fit the data. Since the Ion Proton dataset contains 3 times of the number of benchmark SNVs from Illumina MiSeq and also is enriched with SNVs of ≤ 1 % allele frequency, we mainly focused on Ion Proton data set for model development and evaluation. To test the generalizability of the PSEM, we further trained and evaluated it on Illumina MiSeq dataset.

### Benchmarks overview and comparison

Two sets of designed benchmarks targeting low-frequency SNVs from both Ion Proton and Illumina MiSeq [15] sequencing technologies were included. The details of these 2 datasets are described in Methods section and Additional files 2 and 3. Briefly, the Ion Proton training benchmark is the sequencing data from a single individual with known genotypes, while the test benchmark was designed to mimic the paired normal-tumor design for somatic SNVs identification applications. The Illumina MiSeq benchmark data were generated by mixing 4 individuals at 4 different percentages and then permuted the mixing percentage assignment 4 times to generate 4 calibration datasets – CAL_A, CAL_B, CAL_C and CAL_D. Since the 4 calibration data sets were generated with the same procedures, without loss of generality, we used CAL_A as training benchmark and treated the others as testing benchmark.

**Fig. 1:**
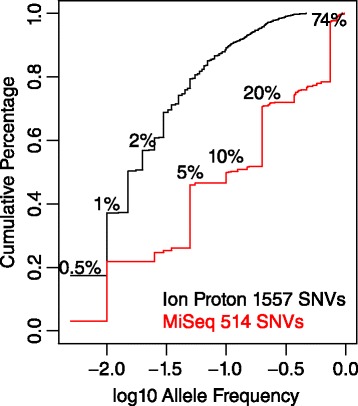
Allele frequency composition of Ion Proton and Illumina MiSeq testing benchmark SNVs

Comparing the two testing benchmarks, Ion Proton contains a total of 1557 somatic SNVs while Illumina MiSeq contains 514 SNV – mixed allele frequency pairs, with 175 unique SNVs. More importantly, Ion Proton benchmark was designed to comprehensively characterize the SNV caller performance on close to sequencing error allele frequencies, thus it is enriched with SNVs of < = 3 % allele frequencies, with 0.5 % as the lowest targeted frequency. Plotting the cumulative percentages of SNV numbers at different allele frequencies (Fig. 1) from the two test benchmarks, it is clear the major components of Ion Proton benchmark SNV allele frequencies are at 0.5 %, 1 %, 2 % to 5 %, followed by continuous frequencies until 46 %, the maximum somatic SNV frequency designed in the dataset. Whereas MiSeq data set includes roughly equal percentages of SNVs at 4 discrete allele frequency levels.

**Fig. 2:**
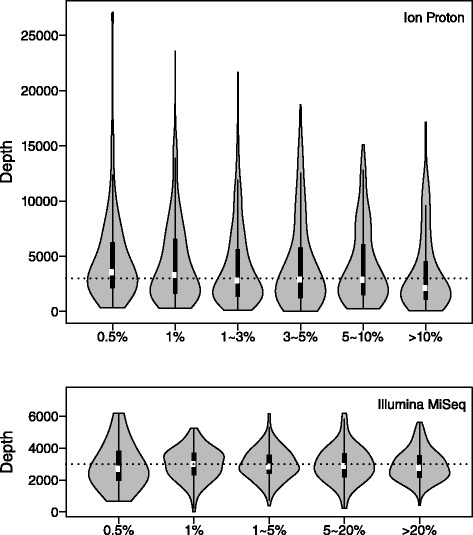
SNV loci depth distribution by allele frequency for Ion Proton and Illumina MiSeq. The dashed lines show the 3000x depth

Except for allele frequency composition, sequencing depth is also a crucial factor affecting the performances of the SNV callers, especially at the low-frequency ranges. The average depth for Ion Proton sequencing testing benchmark is about 4000x and about 1500x for MiSeq. In addition, despite the amplicon-based capture assay was applied on benchmark datasets from both technologies, the evenness of the depth across the targeted regions is different. When comparing the depth on known testing benchmark SNV loci of two technologies (Fig. 2), the depth distribution for Ion Proton is skewed while the distribution profile for Illumina MiSeq data displays a bell shape. Further, the average depth at SNV loci from both benchmarks are around 3000x, despite the much higher overall depth for Ion Proton. Thus, we speculate lowered recall for some Ion Proton benchmark SNVs, particularly for the ≤ 1 % ones, the identifiable power of which are more sensitive to the depth and read count number sampling variances.

### Candidate distributions selection

**Fig. 3:**
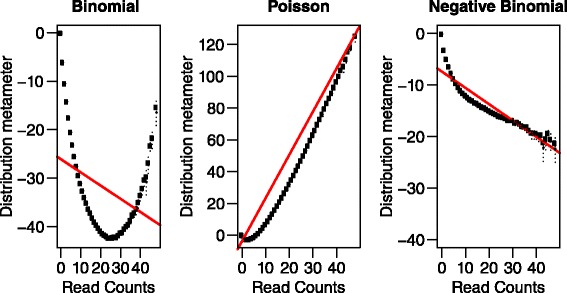
Distplot on binomial, Poisson and negative binomial distributions. The y-axis is the distribution metameter calculated by the method distplot used. The open points show the observed count metameters; the filled points show the confidence interval centers and the dashed lines show the confidence intervals for each point. 95 % confidence interval is used

To model error rate based on count data, 3 most common distribution choices are binomial, Poisson and negative binomial (NB) distributions. We applied a graphical exploratory plot – distplot [16–18] on the model response – number of reads containing non-reference bases – to get visual intuition about the overall fit of response data on different distributions. Intuitively, if an assumed distribution fits the data well, the data points should follow a straight line determined by the distribution metameters. As shown in Fig. 3, the obvious curve for binomial distribution plot suggests binomial distribution is not appropriate. The Poisson and NB plots show better agreement with the straight line although both curves deviate more from the straight line when the x-axis approaches 0. Tabulating the percentages of zero in the model responses show for Ion Proton training dataset, 85 % is 0 while 80 % for MiSeq training data. Thus zero-inflated models should be considered. In the modeling step, we included Poisson, NB and their zero-inflated counterparts (zero-inflated Poisson [19] or ZIP and zero-inflated negative binomial [20] or ZINB) as the candidate distributions under generalized linear model (GLM) framework.

### Comparing the goodness-of-fit of different distributions

9 genomic sequence context covariates, totaling 24 degrees of freedom, were included in the GLM models (Methods and Additional file 4). Since ZIP and ZINB GLM require covariates for both the ‘zero’ and ‘count’ parts, the same covariates were provided for both, resulting in doubled degrees of freedom of those included in Poisson and NB GLM.

To compare the goodness-of-fit of models based on different distributions, we used Vuong’s non-nested hypothesis test [21]. BIC-corrected Vuong z-statistic was used to impose stronger penalty on additional parameters. The pairwise comparison results are summarized in Table 1. Poisson distribution GLM is treated as the reference distribution to compare to, given its simple configuration. As expected, NB GLM is superior to Poisson GLM, since NB models dispersion of the data, and this is also supported by dispersion test [22] (z = 68.5881, *p* value < 2.2e-16). The necessity of modeling zero-inflation is supported by the Vuong’s test comparing ZIP with Poisson GLM. When comparing ZIP with NB, NB fits the data better. However, it is worth noting the evidence of superiority – the absolute value of BIC-corrected Vuong z-statistic – is much smaller than the other tests. The merit of considering both dispersion and zero-inflation is further emphasized by the comparisons of ZIP with ZINB and NB with ZINB. In conclusion, based on Vuong’s test, for Ion Proton sequencing dataset, the most appropriate distribution is ZINB, followed by NB, ZIP and Poisson.

**Table 1:** Vuong’s non-nested tests on 4 distributions applied to Ion Proton training data

Model 1	Model 2	Vuong z-statistic BIC-corrected	Hypothesis	*P* value
Poisson	NB	−122.67	model2 > model1	<2.22e-16
Poisson	ZIP	−143.73	model2 > model1	<2.22e-16
NB	ZIP	36.81	model1 > model2	<2.22e-16
ZIP	ZINB	−92.16	model2 > model1	<2.22e-16
NB	ZINB	−119.51	model2 > model1	<2.22e-16

### Performance evaluation on Ion Proton testing benchmark

We first evaluated the overall precision and recall values of all models on the test benchmark. From Table 2, it is observed the Poisson GLM achieves the highest recall while ZINB GLM has the highest precision. F1 score, the harmonic mean of precision and recall, is used to evaluate the overall performance. The conclusion from F1 score is consistent with that of Vuong’s test, with ZINB performs the best, followed by NB, ZIP and Poisson GLM. However, the precision values listed in Table 2 are lower than the ones reported previously [7, 14, 15]. There are 2 major reasons: 1. the Ion Proton test benchmark dataset is designed to enrich with low-frequency SNVs, with 68.9 % of all SNVs of allele frequency < = 3 %, in which 17.3 % at 0.5 % frequency and 19.8 % at 1 % frequency, whereas the majority of previous studies focused on SNVs of > = 5 % allele frequency; 2. one popular paradigm of SNV calling is a two-step procedure, first generating SNV candidates and then applying multiple sequencing quality filters to refine the SNV call. The PSEM aims to efficiently recover high quality SNV candidates to facilitate the filtering step, thus it is only fair to compare the performance of PSEM with other candidate generating methods. The result from VarScan2 before applying sequencing quality filters was included in Table 2. It is evident that except for Poisson GLM, the other methods outperformed VarScan2 in both recall and precision. Therefore, choosing appropriate statistical modeling method enables us to recover more true SNVs without any loss of precision in candidate generating step.

**Table 2:** Overall performance comparison for Ion Proton testing benchmark

	Poisson	NB	ZIP	ZINB	VarScan2
Recall	0.98	0.89	0.95	0.90	0.83
Precision	0.25	0.62	0.54	0.71	0.53
F1 Score	0.40	0.73	0.69	0.79	0.65

**Fig. 4:**
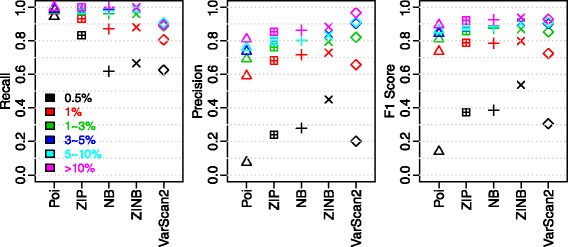
Performance by allele frequency summary on Ion Proton testing benchmark

Next, for all distributions, we explored the performance profiles on different allele frequencies. As shown in Fig. 4, the well-separated F1 score levels clearly show that SNVs of lower allele frequencies are more difficult to identify, no matter what distributions were used. In addition, the significant separation of 0.5 % from the other allele frequencies indicate the detection limit is around 0.5 % under current sequencing platform and depth. Meanwhile, the power of appropriate modeling is evident when comparing the performances of all distributions on SNVs of 0.5 % allele frequency. Relative to Poisson GLM, considering either zero-inflation or dispersion boosted the F1 score by about 0.2 at 0.5 %, while considering both by ZINB further increased F1 score by about 0.1. Interestingly, compared with the second best model – NB GLM, both precision and recall increased in ZINB GLM, which pinpoints the necessity of modeling zero-inflation to derive more accurate error rates estimation. Furthermore, for SNVs with allele frequency greater than 1 %, the average recall is 97.5 % with 82.3 % average precision for ZINB GLM. To summarize, the performance evaluation results on low-frequency SNV identification also support the conclusion from Vuong’s non-nested test, with ZINB being the most appropriate model. Further, the necessity of modeling both dispersion and zero-inflation is exemplified by the much-elevated performance at close to sequencing error rate allele frequency, which is important for pushing down the detection limit of low-frequency SNV callers.

### Application of ZINB PSEM on Illumina MiSeq data

To evaluate the generalizability and adaptiveness of the GLM based PSEM, the same modeling strategies were applied to the Illumina MiSeq sequencing data sets. The same genomic sequence context features from Ion Proton modeling were applied to the Illumina MiSeq CAL_A dataset. Similar to the analysis on Ion Proton data set, paired Vuong’s non-nested hypothesis tests were conducted on the 4 candidate distributions, with details summarized in Additional file 5. The test conclusions remained the same except for the NB (model 1) and ZIP (model 2) comparison, where the BIC-corrected Vuong z-statistic is −0.47 resulting in *p* value = 0.318. Therefore the goodness-of-fit for these two distributions on MiSeq dataset are not significantly different.

Despite similar statistical modeling schema can be readily generalized to Illumina MiSeq data set, Illumina MiSeq and Ion Proton sequencers differ significantly in terms of sequencing chemistry. The former is based on sequencing-by-synthesis (SBS) that relies on high-resolution optic systems, whereas the latter is based on Ion semiconductor sequencing where no modified nucleotides or optics are required. The differences in sequencing mechanisms make Ion Proton sequencers run faster but are prone to homopolymer related errors. Comparing the NB GLM regression coefficients on both datasets (Additional file 6), homopolymer related features significant in Ion Proton data set regression are either insignificant (hmer_len, hmer_dist) or show opposite effect (hmer_op, hmer_den) on the error rate. The same trend was also observed in ZIP and ZINB models comparing Ion Proton with Illumina MiSeq (Additional files 7, 8, 9 and 10).

**Fig. 5:**
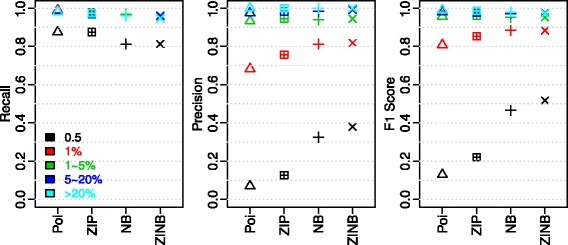
Performance by allele frequency summary on Illumina MiSeq testing benchmark

To evaluate whether the differences in GLM coefficients affect the performance profiles on various allele frequencies, we applied the 4 GLM models trained on CAL_A to the other 3 calibration datasets and conducted the recall, precision and F1 score analyses by allele frequency on the combined dataset. As shown in Fig. 5, similar to the Ion Proton data set, SNVs of lower allele frequencies are more difficult to identify. However, when comparing the performances of ZIP with NB GLM on 0.5 % ~ 1 % allele frequency, different from Ion Proton dataset, NB demonstrated a much higher F1 score compared with ZIP. A closer look at the performance profiles shows the noticeable drop in recall comparing NB with ZIP in Ion Proton is absent in MiSeq data. Examination on the benchmark SNVs missed by NB but recovered by ZIP showed lower depth for the missed ones. While the absent of recall drop in MiSeq is due to its relatively even depth contrast to the Ion Proton dataset (Fig. 2). For SNVs with > 1 % allele frequency, the F1 scores are all greater than 0.9 and clustered together for all distributions.

Comparing with the results from UDT-Seq [15], which reported approximately 90 % recall and >95 % precision (no specific number was given, the precision was inferred by the precision for the other data UDT-Seq tested - Illumina GAII benchmark data at 1500x depth), ZINB GLM demonstrates higher overall recall (95.1 %) and high precision (93.4 %).

## Discussion

The PSEM model aims to predict the position specific error rates associated with various genomic sequence contexts, under which the specific sequencing technology is prone to error. Based on publications evaluating features associated with sequencing errors and experiences from our previous effort, 9 types of significant features are considered. With the features fixed, using GLM, we evaluated the appropriateness of distributions with different mean – variance relationships and the ability to consider zero-inflation. Consistent with the computational tool EdgeR [23] for RNA-Seq data, we found the ability to model over-dispersion by NB distribution necessary for DNA-Seq data as well. Additionally, for DNA-Seq erroneous read counts modeling, zero-inflation is also a key factor for accurate prediction and inference. The much-elevated F1 score for 0.5 % allele frequency SNVs as well as the highest overall performance by ZINB GLM highlighted the importance of choosing suitable statistical models. Moreover, comparing with VarScan2, which conducts the Fisher’s exact test for each targeted location on paired normal-tumor sequencing data, the significance of applying the correct reference error model is exemplified by higher recalls as well as precisions for 0.5 % and 1 % frequency SNVs. In theory, for low frequency SNV loci, VarScan2 treated the sequencing reads with non-reference bases from normal as the background error, which is essentially point estimation based on one location. Whereas PSEM collectively considers all loci with similar context features and thus is able to generate more accurate error estimation.

The evaluation of PSEM modeling on Illumina MiSeq dataset and the performance comparison with Ion Proton dataset show the generality of the PSEM framework as well as its adaptiveness to different technologies. Moreover, except for the established importance of choosing appropriate statistical model, the sequencing depth evenness is also an important factor affecting low-frequency SNVs calling performances.

The current GLM-based PSEM framework only considers 9 types of genome sequence context features. To further improve the performances, more informative features associated with sequencing errors should be included and tested. In addition, from the modeling aspect, exploration of the potential to further increase the performances by applying more sophisticated computational models are desired. To better understand its generalizability and adaptiveness, tests on other sequencing technologies, such us SOLiD and Complete Genomics, are necessary. Besides, since the capture assay for the two benchmarks is amplicon-based, hybridization-based approach should be tested to compare the performance profiles.

Differentiating low frequency SNVs from sequencing artifacts is the key for identifying SNVs at frequencies close to sequencing error rates. Our PSEM approach tried to push the limit toward the sequencing error rates. Based on the analyses on benchmarks from standard sequencing protocols and the given sequencing depth, we speculate the detection limit is around 0.5 % on the regions covering all exons of hundred of genes, with a total size up to millions of bases. However, with high accuracy sequencing protocols, such as duplex sequencing [24] and ultra-deep target enrichment assay [25], the researchers reported identification of SNVs around 0.1 % on a single gene scale. Despite the promising results, more efforts to make such protocols applicable on larger regions are required for broader applications.

## Conclusion

Our method enables sensitive detection of low-frequency single nucleotide variants across different sequencing platforms down to 0.5 % frequency. Thus will facilitate research and clinical applications such as pooled sequencing, cancer early detection, prognostic assessment, metastatic monitoring, and relapses or acquired resistance identification.

## Methods

### Overall workflow

For position specific error model training, we used the invariant loci from training benchmark. Genomic sequence context features were extracted for each locus and then fed to the generalized linear models using 4 different distributions. Then testing benchmark paired tumor and normal sequencing data went through the PSEM and the candidate SNVs were derived. Additional file 11 provides a diagram illustrating this procedure. In the following method section, we first introduced the benchmark datasets from both Ion Proton and Illumina MiSeq. Then we described the application of generalized linear models for PSEM. Last, we described the performance evaluation metrics.

### Benchmark dataset

Both Ion Proton and Illumina MiSeq datasets were generated from amplicon-based targeted sequencing.

The targeted region for Ion Proton datasets included all exons of 409 known cancer-related genes, totaling about 1.7 million bases covered by about 16,000 amplicon primer pairs from Ion AmpliSeq™ Comprehensive Cancer Panel. The training benchmark is the DNA sequencing data of NA11993. The testing benchmark mimic the paired normal-tumor design, where the normal sample is the DNA sequencing data of NA12878 while tumor sample is a mixture of 17 individuals from 1000 Genomics Project plus NA12878. The mixing percentage assignment is listed in Additional file 2. The sequencing data were aligned with TMAP from Torrent Suite software. Reads with mapping quality less than 40 were filtered out.

The length of targeted regions for Illumina MiSeq datasets is 23.2 kb, covered by 158 amplicons. The design details can be found in the paper [15] and Additional file 3. The raw reads were downloaded from NCBI Short Read Archive (SRP009487.1) and processed as the paper described. Reads with mapping quality less than 30 were filtered out.

### Generalized linear models

The details of the 9 genomic sequence contexts considered in GLM were summarized in Additional file 4. Briefly, general contexts including substitution types, immediate upstream and downstream bases, GC content, and homopolymer related features: whether the locus is within a homopolymer, the closest homopolymer length, the distance to the closest homopolymer, the local homopolymer base percentages and whether the alternative base is the same as the immediate upstream or downstream base are considered. These 9 features are the covariates included in the GLMs.

The Poisson GLM for erroneous sequencing read counts with log link function is expressed in eq. (1), where *N*_*s*,*b*,*l*_ is the observed number of erroneous reads for strand *s* (forward or reverse) with alternative base *b* (three possible values other than the reference) at location *l*, *λ*_*s*,*b*,*l*_ represents the expected mean for *N*_*s*,*b*,*l*_, *c*_*s*,*b*,*l*_ is the vector of genomic sequence context covariates, and *β* is the vector of fitted coefficients. The sequencing depth for strand *s* at location *l* is treated as the offset.

1 $$ \log \left({\lambda}_{s,b,l}\right)= \log \left(E\left({N}_{s,b,l}\Big|{\boldsymbol{c}}_{s,b,l}\right)\right)= \log \left({d}_{s,l}\right)+{\boldsymbol{\beta}}^{\boldsymbol{\hbox{'}}}{\boldsymbol{c}}_{s,b,l} $$

The negative binomial distribution GLM with log link function can be expressed in eq. (2), where *μ*_*s*,*b*,*l*_ represents the expected mean for *N*_*s*,*b*,*l*_ and *θ* is the dispersion parameter (the shape parameter of the gamma mixing distribution). The mean *E*(*N*_*s*,*b*,*l*_) = *μ*_*s*,*b*,*l*_ and variance *VAR*(*N*_*s*,*b*,*l*_) = *μ*_*s*,*b*,*l*_ + *θμ*_*s*,*b*,*l*_^2^ can be estimated from GLM shown below.

2 $$ \log \left({\mu}_{s,b,l}\right)= \log \left(E\left({N}_{s,b,l}\Big|{\boldsymbol{c}}_{s,b,l}\right)\right)= \log \left({d}_{s,l}\right)+{\boldsymbol{\beta}}^{\boldsymbol{\hbox{'}}}{\boldsymbol{c}}_{s,b,l} $$

The zero-inflated Poisson distribution can be written as:

3 $$ P\left({N}_{s,b,l}={n}_{s,b,l}\Big|{\pi}_{s,b,l},\ {\lambda}_{s,b,l},\theta \right)=\Big\{\begin{array}{c}\hfill {\pi}_{s,b,l}+\left(1-{\pi}_{s,b,l}\right) Pois\left({\lambda}_{s,b,l};0\right)\kern3.5em  if\kern0.5em {n}_{s,b,l}=0\hfill \\ {}\hfill \left(1-{\pi}_{s,b,l}\right) Pois\left({\lambda}_{s,b,l};{n}_{s,b,l}\right)\kern5.5em  if\kern0.5em {n}_{s,b,l}>0\hfill \end{array} $$

Parameters of the zero-inflated Poisson distribution (3) can be estimated by generalized linear model as shown in (4), where *z*_*s*,*b*,*l*_ is the vector of genomic sequence context covariates for the zero part, and *γ* is the vector of fitted coefficients.

4 $$ \mathrm{logit}\left(\frac{\pi_{s,b,l}}{1-{\pi}_{s,b,l}}\right)=\boldsymbol{\gamma} \boldsymbol{\hbox{'}}{\boldsymbol{z}}_{s,b,l} $$

$$ \log \left({\lambda}_{s,b,l}\right)={\boldsymbol{\beta}}^{\boldsymbol{\hbox{'}}}{\boldsymbol{c}}_{s,b,l} $$

The zero-inflated negative binomial distribution can be written as:

5 $$ P\left({N}_{s,b,l}={n}_{s,b,l}\Big|{\boldsymbol{c}}_{s,b,l},\ {\boldsymbol{z}}_{s,b,l}\right)=\Big\{\begin{array}{c}\hfill {\pi}_{s,b,l}+\left(1-{\pi}_{s,b,l}\right)NB\left({\mu}_{s,b,l},\ \theta; 0\right)\kern2.75em  if\kern0.5em {n}_{s,b,l}=0\hfill \\ {}\hfill \left(1-{\pi}_{s,b,l}\right)NB\left({\mu}_{s,b,l},\theta; {n}_{s,b,l}\right)\kern4.75em  if\kern0.5em {n}_{s,b,l}>0\hfill \end{array} $$

Parameters of the zero-inflated negative binomial distribution (5) can be estimated by generalized linear model as shown in (6).

6 $$ \mathrm{logit}\left(\frac{\pi_{s,b,l}}{1-{\pi}_{s,b,l}}\right)=\boldsymbol{\gamma} \boldsymbol{\hbox{'}}{\boldsymbol{z}}_{s,b,l} $$

$$ \log \left({\mu}_{s,b,l}\right)={\boldsymbol{\beta}}^{\boldsymbol{\hbox{'}}}{\boldsymbol{c}}_{s,b,l} $$

A location with a certain alternative base is called as a candidate SNV if the numbers of reads from both strands are significantly greater than the predicted error rates. The *p* values were corrected using Benjamini–Hochberg procedure. The corrected *p* value cut-off is 0.01.

### Performance evaluation measurements

Precision, recall and F1 score are defined below.

$$ precision=\frac{number\  of\  recovered\  benchmark\  SNVs}{number\  of\  predicted\  SNVs} $$

$$ recall=\frac{number\  of\  recovered\  benchmark\  SNVs}{expected\  number\  of\  benchmark\  SNVs} $$

$$ F1=2*\frac{precision* recall}{precision+ recall} $$

For Ion Proton dataset, loci with at least 5 reads supporting alternative base are included in the evaluation. For Illumina MiSeq dataset, filter 2 used by UDT-Seq was applied which requires > = 0.2 % frequency for alternative bases. However, the other filters were not used. We relied on the PSEM framework to properly address sequencing problems, for example, uneven depth and local sequence context induced errors.

## Additional files

Additional file 1: Summary of major somatic callers, their methods of detecting SNV candidates and the limitations. (PDF 60 kb) Additional file 2: Ion Proton testing benchmark design. (PDF 58 kb) Additional file 3: Illumina MiSeq benchmark design. (PDF 58 kb) Additional file 4: Genomic sequence context features used in GLM. (PDF 71 kb) Additional file 5: Vuong’s non-nested test on 4 distributions applied to Illumina MiSeq training data. (PDF 65 kb) Additional file 6: Negative binomial GLM coefficients for Ion Proton and Illumina MiSeq training datasets. (PDF 71 kb) Additional file 7: Zero-inflated Poisson GLM coefficients for Ion Proton training datasets. (PDF 69 kb) Additional file 8: Zero-inflated Negative Binomial GLM coefficients for Ion Proton training datasets. (PDF 69 kb) Additional file 9: Zero-inflated Poisson GLM coefficients for Illumina MiSeq training datasets. (PDF 71 kb) Additional file 10: Zero-inflated Negative Binomial GLM coefficients for Illumina MiSeq training datasets. (PDF 70 kb) Additional file 11: Overall workflow. This diagram illustrates the training and testing steps. The training data and the position specific error model derived from it are highlighted with dashed lines. After training, testing benchmark paired normal and tumor samples go through the PSEM model and the candidate SNVs are derived. (PDF 115 kb)
