# Supplementary material for: Statistical modeling for sensitive detection of low-frequency single nucleotide variants
Source: BMC Genomics. 2016 Aug 22;17(Suppl 7):514. doi: 10.1186/s12864-016-2905-x (PMC5001245; doi:10.1186/s12864-016-2905-x)
Supplement: Additional file 1: — Summary of major somatic callers, their methods of detecting SNV candidates and the limitations. (PDF 60 kb) [file 12864_2016_2905_MOESM1_ESM.pdf]

Additional file 1 - Summary of major somatic callers, their methods of detecting SNV candidates and the limitations

Mutation Callers	Candidate Identification Method	Limitation
VarScan2	Fisher's exact test.	Single point estimation of sequencing error for each locus.
Strelka	Bayesian approach that jointly consider normal SNV genotype and continuous tumor SNV allele frequencies.	At each locus, assuming equal error rate for the 3 possible alternatives.
MuTect	Candidate SNV detection using log odds score to differentiate sequence error from true variants.	Assuming all substitution errors are equally likely.
UDT-Seq	Tabulate the error rates based on multiple context features, then apply binomial model to detect candidates.	Tabulation based method has limited ability to incorporate larger number of features. Distributions other than binomial can better deal with count data.
