# Supplementary material for: Statistical modeling for sensitive detection of low-frequency single nucleotide variants
Source: BMC Genomics. 2016 Aug 22;17(Suppl 7):514. doi: 10.1186/s12864-016-2905-x (PMC5001245; doi:10.1186/s12864-016-2905-x)
Supplement: Additional file 2: — Ion Proton testing benchmark design. (PDF 58 kb) [file 12864_2016_2905_MOESM2_ESM.pdf]

Additional file 2 - Ion Proton testing benchmark design

Individual NA12878 was used as the ‘normal’ sample.

ID	Mixing Percent
NA18507	1%
NA18853	1%
NA18526	1%
NA19239	1%
NA19238	1%
NA19092	1%
NA18870	2%
NA18502	2%
NA18871	2%
NA18501	2%
NA18987	3%
NA12872	3%
NA18622	3%
NA12751	6%
NA07000	6%
NA18965	8%
NA12750	10%
**NA12878**	**46%**
