# Supplementary material for: Statistical modeling for sensitive detection of low-frequency single nucleotide variants
Source: BMC Genomics. 2016 Aug 22;17(Suppl 7):514. doi: 10.1186/s12864-016-2905-x (PMC5001245; doi:10.1186/s12864-016-2905-x)
Supplement: Additional file 3: — Illumina MiSeq benchmark design. (PDF 58 kb) [file 12864_2016_2905_MOESM3_ESM.pdf]

Additional file 3 - Illumina MiSeq benchmark design

ID	CAL_A	CAL_B	CAL_C	CAL_D
NA12156	1%	5%	20%	74%
NA12878	5%	20%	74%	1%
NA18507	20%	74%	1%	5%
NA19240	74%	1%	5%	20%
