# Supplementary material for: Statistical modeling for sensitive detection of low-frequency single nucleotide variants
Source: BMC Genomics. 2016 Aug 22;17(Suppl 7):514. doi: 10.1186/s12864-016-2905-x (PMC5001245; doi:10.1186/s12864-016-2905-x)
Supplement: Additional file 4: — Genomic sequence context features used in GLM. (PDF 71 kb) [file 12864_2016_2905_MOESM4_ESM.pdf]

Additional file 4 - Genomic sequence context features used in GLM

Features	Definition	Covariates
NA	Intercept only model, containing the baseline for each feature. Specifically: substitution is A → C, upstream and downstream bases are both A; GC, hmer_dist, hmer_len, hden, hrun_op and alt_up_down_eq are all 0.	Intercept
substitution	Change from reference base to alternative base; there are 12 possible values. A → G means reference base A to alternative base G.	A → G
		A → T
		C → A
		C → G
		C → T
		G → A
		G → C
		G → T
		T → A
		T → C
		T → G
upstream base	Immediate upstream base (4 possible values: A,C,G,T).	C
		G
		T
downstream base	Immediate downstream base (4 possible values: A,C,G,T).	C
		G
		T
GC content	Percent of GC bases within a 101 base window that extends 50 nucleotides both upstream and downstream.	GC
distance to the closest homopolymer base	Number of nucleotides to the closest base of the homopolymer within a window that extends 15 bases both upstream and downstream (possible values 0 to 13, 15 for no homopolymer within the window).	hmer_dist
length of the closest homopolymer	Length of the closest homopolymer within a window that extends 15 bases both upstream and downstream (possible values 0, 3, 4 to 31).	hmer_len
homopolymer bases percentage	Fraction of bases within a 31 bases window that are in homopolymers (possible values 0, 3/31, 4/31 to 1). The window extends 15 bases both upstream and downstream.	hmer_percent
overlap with homopolymer	Whether the locus of interest is within a homopolymer, 1 means yes, 0 means no.	hmer_op
upstream or downstream base shift	Whether the alternative base is the same as the immediate upstream or downstream base, 1 means yes, 0 means no.	alt_up_down
