# Supplementary material for: Statistical modeling for sensitive detection of low-frequency single nucleotide variants
Source: BMC Genomics. 2016 Aug 22;17(Suppl 7):514. doi: 10.1186/s12864-016-2905-x (PMC5001245; doi:10.1186/s12864-016-2905-x)
Supplement: Additional file 5: — Vuong’s non-nested test on 4 distributions applied to Illumina MiSeq training data. (PDF 65 kb) [file 12864_2016_2905_MOESM5_ESM.pdf]

Additional file 5 - Vuong's non-nested test on 4 distributions applied to Illumina MiSeq training data

Model 1	Model 2	Vuong z-statistic BIC-corrected	Hypothesis	P value
Poisson	NB	-23.38	model2 > model1	< 2.22e-16
Poisson	ZIP	-21.30	model2 > model1	< 2.22e-16
NB	ZIP	-0.47	model2 < model1	0.31796
ZIP	ZINB	-20.22	model2 > model1	< 2.22e-16
NB	ZINB	-17.44	model2 > model1	< 2.22e-16
