# Supplementary material for: Statistical modeling for sensitive detection of low-frequency single nucleotide variants
Source: BMC Genomics. 2016 Aug 22;17(Suppl 7):514. doi: 10.1186/s12864-016-2905-x (PMC5001245; doi:10.1186/s12864-016-2905-x)
Supplement: Additional file 6: — Negative binomial GLM coefficients for Ion Proton and Illumina MiSeq training datasets. (PDF 71 kb) [file 12864_2016_2905_MOESM6_ESM.pdf]

Additional file 6 - Negative binomial GLM coefficients for Ion Proton and Illumina MiSeq training datasets

	Ion Proton			Illumina MiSeq		
Parameter	Estimate	Standard Error	P value	Estimate	Standard Error	P value
(Intercept)	-10.9331	0.0105	< 2e-16	-1.0700	0.0290	< 2e-16
A → C	-0.1516	0.0072	< 2e-16	0.6922	0.0220	< 2e-16
A → G	1.5093	0.0060	< 2e-16	2.6550	0.0190	< 2e-16
A → T	-0.0352	0.0072	< 2e-16	-0.2219	0.0270	4.26e-16
C → A	-0.1073	0.0075	< 2e-16	-0.3068	0.0290	< 2e-16
C → G	-0.3362	0.0080	< 2e-16	-0.9909	0.0360	< 2e-16
C → T	1.3287	0.0062	< 2e-16	1.8840	0.0200	< 2e-16
G → A	1.2600	0.0062	< 2e-16	1.7870	0.0200	< 2e-16
G → C	-0.2030	0.0077	< 2e-16	-0.7178	0.0330	< 2e-16
G → T	-0.1705	0.0075	< 2e-16	-0.7540	0.0360	< 2e-16
T → A	0.0445	0.0071	< 2e-16	-0.2594	0.0270	< 2e-16
T → C	1.5362	0.0059	< 2e-16	2.7120	0.0190	< 2e-16
up base A	0.1046	0.0037	< 2e-16	0.1640	0.0100	< 2e-16
up base C	0.2316	0.0038	< 2e-16	0.4087	0.0100	< 2e-16
up base G	0.3288	0.0037	< 2e-16	0.5110	0.0100	< 2e-16
down base A	-0.0908	0.0037	< 2e-16	-0.2120	0.0100	< 2e-16
down base C	0.3356	0.0036	< 2e-16	0.3462	0.0090	< 2e-16
down base G	0.1748	0.0037	< 2e-16	0.2600	0.0090	< 2e-16
GC	0.0058	0.0001	< 2e-16	0.0099	0.0003	< 2e-16
hmer_den	0.2994	0.0136	< 2e-16	-0.0798	0.0360	0.028
hmer_op	0.3313	0.0037	< 2e-16	-0.1430	0.0100	< 2e-16
hmer_dist	-0.0137	0.0004	< 2e-16	-0.0009	0.0010	0.4
hmer_len	0.0790	0.0012	< 2e-16	0.0018	0.0030	0.569
alt_up_down	0.7385	0.0026	< 2e-16	0.0760	0.0070	< 2e-16
