# Supplementary material for: Statistical modeling for sensitive detection of low-frequency single nucleotide variants
Source: BMC Genomics. 2016 Aug 22;17(Suppl 7):514. doi: 10.1186/s12864-016-2905-x (PMC5001245; doi:10.1186/s12864-016-2905-x)
Supplement: Additional file 7: — Zero-inflated Poisson GLM coefficients for Ion Proton training datasets. (PDF 69 kb) [file 12864_2016_2905_MOESM7_ESM.pdf]

Additional file 7 - Zero-inflated Poisson GLM coefficients for Ion Proton training datasets

	Ion Proton ZIP Zero			Ion Proton ZIP Count		
Parameter	Estimate	Standard Error	P value	Estimate	Standard Error	P value
(Intercept)	1.9146	0.0171	<.0001	-8.8454	0.0116	<.0001
A → C	0.1445	0.0107	<.0001	-0.0243	0.0090	0.0073
A → G	-2.0633	0.0093	<.0001	0.1400	0.0068	<.0001
A → T	0.1197	0.0106	<.0001	0.0946	0.0089	<.0001
C → A	0.2546	0.0110	<.0001	0.1415	0.0092	<.0001
C → G	0.4922	0.0116	<.0001	0.1091	0.0098	<.0001
C → T	-2.1312	0.0111	<.0001	-0.1127	0.0074	<.0001
G → A	-2.0341	0.0109	<.0001	-0.1449	0.0075	<.0001
G → C	0.4040	0.0112	<.0001	0.1570	0.0094	<.0001
G → T	0.1876	0.0111	<.0001	0.0191	0.0095	0.0440
T → A	0.1497	0.0103	<.0001	0.1971	0.0087	<.0001
T → C	-2.0191	0.0092	<.0001	0.1767	0.0068	<.0001
up base A	-0.0721	0.0063	<.0001	0.0706	0.0040	<.0001
up base C	-0.2825	0.0064	<.0001	0.0834	0.0040	<.0001
up base G	-0.3168	0.0061	<.0001	0.1817	0.0039	<.0001
down base A	0.1466	0.0066	<.0001	-0.0295	0.0042	<.0001
down base C	-0.1925	0.0060	<.0001	0.2428	0.0037	<.0001
down base G	-0.1177	0.0062	<.0001	0.1291	0.0039	<.0001
GC	0.0014	0.0002	<.0001	0.0061	0.0001	<.0001
hmer_den	0.3180	0.0221	<.0001	0.3798	0.0133	<.0001
hmer_op	0.0696	0.0060	<.0001	0.3578	0.0036	<.0001
hmer_dist	0.0320	0.0007	<.0001	0.0099	0.0004	<.0001
hmer_len	0.0332	0.0019	<.0001	0.0918	0.0012	<.0001
alt_up_down	-0.4933	0.0044	<.0001	0.3525	0.0027	<.0001
