# Supplementary material for: Statistical modeling for sensitive detection of low-frequency single nucleotide variants
Source: BMC Genomics. 2016 Aug 22;17(Suppl 7):514. doi: 10.1186/s12864-016-2905-x (PMC5001245; doi:10.1186/s12864-016-2905-x)
Supplement: Additional file 8: — Zero-inflated Negative Binomial GLM coefficients for Ion Proton training datasets. (PDF 69 kb) [file 12864_2016_2905_MOESM8_ESM.pdf]

Additional file 8 - Zero-inflated Negative Binomial GLM coefficients for Ion Proton training datasets

	Ion Proton ZINB Zero			Ion Proton ZINB Count		
Parameter	Estimate	Standard Error	P value	Estimate	Standard Error	P value
(Intercept)	1.1257	0.0242	<.0001	-9.5531	0.0137	<.0001
A → C	0.1630	0.0157	<.0001	-0.0452	0.0133	0.0007
A → G	-15.1084	7.8315	0.0537	0.2005	0.0096	<.0001
A → T	0.2468	0.0152	<.0001	0.1734	0.0131	<.0001
C → A	0.4575	0.0155	<.0001	0.2629	0.0137	<.0001
C → G	0.5520	0.0164	<.0001	0.1351	0.0149	<.0001
C → T	-15.2409	9.4679	0.1075	0.0056	0.0098	0.5662
G → A	-14.9469	8.7844	0.0888	-0.0712	0.0098	<.0001
G → C	0.4937	0.0158	<.0001	0.2405	0.0144	<.0001
G → T	0.3495	0.0158	<.0001	0.1213	0.0138	<.0001
T → A	0.3202	0.0147	<.0001	0.3211	0.0129	<.0001
T → C	-15.0527	7.8142	0.0541	0.2217	0.0096	<.0001
up base A	-0.0263	0.0084	0.0017	0.1111	0.0039	<.0001
up base C	-0.0485	0.0088	<.0001	0.2576	0.0040	<.0001
up base G	0.1272	0.0085	<.0001	0.4031	0.0039	<.0001
down base A	0.0244	0.0086	0.0044	-0.1161	0.0040	<.0001
down base C	0.1622	0.0083	<.0001	0.3995	0.0038	<.0001
down base G	0.0915	0.0085	<.0001	0.2305	0.0040	<.0001
GC	0.0005	0.0003	0.1233	0.0057	0.0001	<.0001
hmer_den	-0.1738	0.0303	<.0001	0.1658	0.0144	<.0001
hmer_op	-0.1482	0.0082	<.0001	0.2612	0.0039	<.0001
hmer_dist	0.0569	0.0010	<.0001	0.0037	0.0004	<.0001
hmer_len	-0.0223	0.0028	<.0001	0.0685	0.0013	<.0001
alt_up_down	-0.7678	0.0060	<.0001	0.4657	0.0028	<.0001
