# Supplementary material for: Statistical modeling for sensitive detection of low-frequency single nucleotide variants
Source: BMC Genomics. 2016 Aug 22;17(Suppl 7):514. doi: 10.1186/s12864-016-2905-x (PMC5001245; doi:10.1186/s12864-016-2905-x)
Supplement: Additional file 9: — Zero-inflated Poisson GLM coefficients for Illumina MiSeq training datasets. (PDF 71 kb) [file 12864_2016_2905_MOESM9_ESM.pdf]

Additional file 9 - Zero-inflated Poisson GLM coefficients for Illumina MiSeq training datasets

	Illumina MiSeq ZIP Zero			Illumina MiSeq ZIP Count		
Parameter	Estimate	Standard Error	P value	Estimate	Standard Error	P value
(Intercept)	-4.7601	0.0792	< 2e-16	-8.2124	0.0357	< 2e-16
A → C	-0.5475	0.0368	< 2e-16	0.2277	0.0303	5.76e-14
A → G	-4.0851	0.0425	< 2e-16	0.4489	0.0260	< 2e-16
A → T	-2.0125	0.1177	< 2e-16	-1.6997	0.0736	< 2e-16
C → A	-0.3830	0.0563	1.02e-11	-0.5424	0.0511	< 2e-16
C → G	0.2114	0.0728	0.00367	-0.7162	0.0694	< 2e-16
C → T	-4.0547	0.0727	< 2e-16	-0.3198	0.0289	< 2e-16
G → A	-4.1014	0.0821	< 2e-16	-0.4230	0.0295	< 2e-16
G → C	-0.3811	0.0759	5.12e-07	-0.9758	0.0690	< 2e-16
G → T	-0.2028	0.0731	0.00552	-0.8586	0.0673	< 2e-16
T → A	-2.2197	0.1292	< 2e-16	-1.8542	0.0745	< 2e-16
T → C	-4.0724	0.0414	< 2e-16	0.5122	0.0259	< 2e-16
up base A	0.1812	0.0342	1.20e-07	0.2259	0.0114	< 2e-16
up base C	0.0145	0.0330	0.6600	0.4132	0.0110	< 2e-16
up base G	0.0292	0.0333	0.3802	0.5447	0.0110	< 2e-16
down base A	-0.0294	0.0332	0.3753	-0.2253	0.0113	< 2e-16
down base C	-0.0892	0.0282	0.00158	0.3286	0.0094	< 2e-16
down base G	-0.1613	0.0284	1.37e-08	0.2072	0.0094	< 2e-16
GC	-0.0046	0.0009	1.35e-06	0.0076	0.0003	< 2e-16
hmer_den	-0.1074	0.1109	0.3329	-0.1332	0.0367	0.000288
hmer_op	0.1045	0.0324	0.00125	-0.0982	0.0110	< 2e-16
hmer_dist	-0.0020	0.0032	0.5241	-0.0008	0.0010	0.4201
hmer_len	0.0054	0.0098	0.5840	0.0027	0.0032	0.3979
alt_up_down	-0.1274	0.0202	2.99e-10	0.0404	0.0068	2.62e-09
