# Supplementary material for: Statistical modeling for sensitive detection of low-frequency single nucleotide variants
Source: BMC Genomics. 2016 Aug 22;17(Suppl 7):514. doi: 10.1186/s12864-016-2905-x (PMC5001245; doi:10.1186/s12864-016-2905-x)
Supplement: Additional file 10: — Zero-inflated Negative Binomial GLM coefficients for Illumina MiSeq training datasets. (PDF 70 kb) [file 12864_2016_2905_MOESM10_ESM.pdf]

Additional file 10 - Zero-inflated Negative Binomial GLM coefficients for Illumina MiSeq training datasets

	Illumina MiSeq ZINB Zero			Illumina MiSeq ZINB Count		
Parameter	Estimate	Standard Error	P value	Estimate	Standard Error	P value
(Intercept)	-4.9463	0.0882	< 2e-16	-8.5006	0.0403	< 2e-16
A → C	-0.5390	0.0425	< 2e-16	0.2516	0.0386	0.0000
A → G	-20.3654	199.8785	0.9188	0.5605	0.0327	< 2e-16
A → T	-2.5739	0.1941	< 2e-16	-1.8410	0.0804	< 2e-16
C → A	-0.3709	0.0652	0.0000	-0.5568	0.0597	< 2e-16
C → G	0.2103	0.0832	0.0115	-0.7645	0.0795	< 2e-16
C → T	-18.8859	160.8217	0.9065	-0.1999	0.0333	0.0000
G → A	-18.9098	175.1755	0.9140	-0.2965	0.0334	< 2e-16
G → C	-0.4230	0.0867	0.0000	-1.0371	0.0765	< 2e-16
G → T	-0.2191	0.0843	0.0093	-0.9073	0.0766	< 2e-16
T → A	-2.9141	0.2342	< 2e-16	-1.9927	0.0787	< 2e-16
T → C	-21.4824	350.3248	0.9511	0.6199	0.0327	< 2e-16
up base A	0.2146	0.0355	0.0000	0.1933	0.0102	< 2e-16
up base C	0.1302	0.0359	0.0003	0.4234	0.0103	< 2e-16
up base G	0.2396	0.0345	0.0000	0.5518	0.0101	< 2e-16
down base A	-0.0373	0.0357	0.2954	-0.2143	0.0102	< 2e-16
down base C	0.0437	0.0338	0.1959	0.3563	0.0096	< 2e-16
down base G	-0.1327	0.0341	0.0001	0.2369	0.0096	< 2e-16
GC	-0.0124	0.0011	< 2e-16	0.0069	0.0003	< 2e-16
hmer_den	-0.2782	0.1289	0.0309	-0.1402	0.0371	0.0002
hmer_op	0.0699	0.0358	0.0506	-0.1256	0.0104	< 2e-16
hmer_dist	0.0051	0.0038	0.1842	0.0003	0.0011	0.7654
hmer_len	0.0075	0.0116	0.5139	0.0029	0.0033	0.3820
alt_up_down	0.0171	0.0247	0.4879	0.0825	0.0070	< 2e-16
