# Supplementary figures and images for: Statistical modeling for sensitive detection of low-frequency single nucleotide variants
Source: BMC Genomics. 2016 Aug 22;17(Suppl 7):514. doi: 10.1186/s12864-016-2905-x (PMC5001245; doi:10.1186/s12864-016-2905-x)

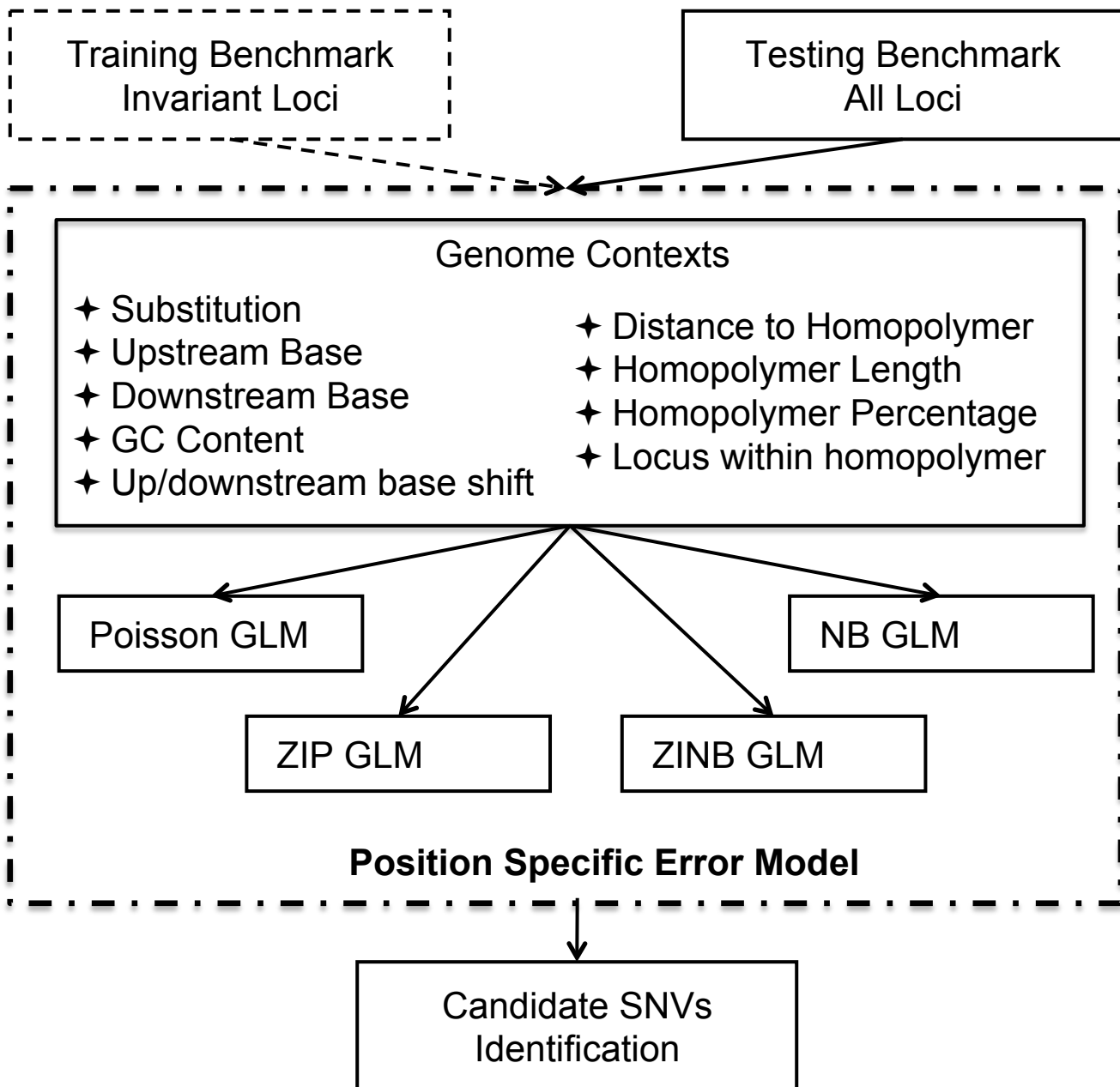

Supplement: Additional file 11: — Overall workflow. This diagram illustrates the training and testing steps. The training data and the position specific error model derived from it are highlighted with dashed lines. After training, testing benchmark paired normal and tumor samples go through the PSEM model and the candidate SNVs are derived. (PDF 115 kb) [file 12864_2016_2905_MOESM11_ESM.pdf]
